# Effect of Strengthening Location on Seismic Performance of Masonry Domes Retrofitted with Composite Material

**DOI:** 10.3390/polym17212921

**Published:** 2025-10-31

**Authors:** Tulin Celik, Ali Ural

**Affiliations:** Department of Civil Engineering, Aksaray University, Aksaray 68100, Turkey; aural@aksaray.edu.tr

**Keywords:** masonry dome, CFRP, repair and strengthening, experiment and analysis

## Abstract

In this study, the effectiveness of a carbon fiber-reinforced polymer (CFRP) system applied to different regions for the strengthening of historical masonry domes was investigated, and the effects of the CFRP material on the structural performance of different regions were evaluated. One model served as the reference and did not include any reinforcement. In the other three models, reinforcement was applied by wrapping the CFRP around only the skirt region (EPS), only the drum region (EPD), and both the skirt and drum regions (EPSD). The effects of these reinforcement methods on the structural performance were analyzed through experimental tests simulating earthquake effects applied to the dome body wall region. The experimental findings were compared with numerical modeling results obtained using LUSAS V19.0 finite element software, and the overall effectiveness of the reinforcement methods was evaluated holistically. The results show that applying CFRP reinforcement only to the drum (rim) region provides the highest bearing capacity and is the most effective solution in terms of structural performance.

## 1. Introduction

Historical structures are original works that reflect the architectural features of the period in which they were built and have survived from the past to the present, preserving cultural heritage. Over time, these structures may have deteriorated due to various external factors, threatening both their structural integrity and historical value. Therefore, the repair and reinforcement of historical structures must be carried out carefully, without compromising their authenticity, and in accordance with conservation principles. This process is not only a technical intervention but is also crucial for ensuring the healthy transmission of cultural heritage to future generations.

For reinforcement applications, the structural condition of the load-bearing system should be evaluated first, followed by a comprehensive analysis of the building materials and ground conditions. However, many historical structures are renewed or strengthened without sufficient engineering knowledge or detailed analysis. This can negatively affect both the safety and historical value of structures. The main causes of damage to historical structures include weak ground conditions, natural disasters, material deterioration, construction errors, and wars. The primary goal of addressing such damage is to protect the load-bearing integrity of a structure and maintain its overall functionality [1].

There is a significant gap in the literature regarding the effective use of a carbon fiber-reinforced polymer (CFRP) system in masonry domes, and the most appropriate application of this system to different structural regions. This study aims to fill that gap by providing practical guidance on how the CFRP technique can be efficiently applied to strengthen historical structures. The findings are intended to assist professionals, including engineers, architects, and conservationists, in selecting the most suitable methods for similar projects and to contribute to the sustainable protection of cultural heritage.

The preservation of historical structures has long been a major focus in structural engineering and has been the subject of numerous studies examining their behavior under various loading conditions, including dead loads, wind, and earthquakes [2,3,4,5,6,7,8,9,10,11,12,13,14,15,16,17,18]. Among these structures, masonry domes hold particular importance due to their unique geometric forms and material properties. These structural features not only define the architectural identity of the domes but also directly influence their structural behavior, making them a critical subject of research in the development of evaluation and strengthening techniques.

Foraboschi [19] highlighted that the load-bearing behavior of masonry domes is primarily influenced by their structural geometry and the arrangement of units, with the material’s strength playing a secondary role. Due to the tensile stresses induced by environmental factors, masonry domes tend to behave more like a network of interconnected arches than as continuous shell systems. In recent years, considerable research has focused on strengthening these structures using modern composite materials. Tuhta et al. [20] demonstrated that masonry domes reinforced with carbon fiber-reinforced polymer (CFRP) fabrics exhibited significant improvements in their dynamic properties, particularly in terms of natural period and frequency, thereby enhancing the overall structural safety. Similarly, Al-Saigh and Al-Mahaidi [21] investigated CFRP applications for repairing damage to the dome of the Al-Abbas bin Ali mausoleum, reporting the effective restoration of structural integrity following damage from explosions and heavy impacts. Hrasnica and Medic [22] further confirmed the effectiveness of CFRP systems for strengthening delicate and slender stone elements, such as minarets, achieving reinforcement without compromising the aesthetic values. In a seismic analysis of the Soltaniyeh Dome, Feizolahbeigi [18] identified minarets as the most vulnerable and critical components, highlighting the importance of targeted regional strengthening strategies.

Despite these advances, a notable gap remains in the literature regarding the strengthening of masonry domes with FRP materials. Bayraktar et al. [23] provided a comprehensive review of the static and dynamic behavior of historical masonry domes and evaluated various reinforcement techniques, including steel tensioners, FRP/CFRP sheets, fiber-reinforced cementitious matrix (FRCM) composites, and expansive mortars. Their study focused particularly on the crack patterns, collapse mechanisms, and the compatibility of these interventions with conservation principles. However, they emphasized the scarcity of experimental investigations under strong ground motion scenarios, highlighting the need for further research.

In this context, Gandolfi et al. [24] developed a low-cost and practical nonlinear analysis method for FRP-reinforced masonry domes, effectively capturing the increases in the load-carrying capacity and energy dissipation. Their model, validated against experimental data, offers direct applicability in engineering practice. Fraternali et al. [25] introduced an innovative tensegrity-based optimization method for domes and vaults strengthened with FRP/FRCM, optimizing the internal force distributions to ensure balanced compression in the masonry units and tension in the composite elements, resulting in lightweight, crack-compatible reinforcement systems.

Experimental studies have also provided critical insights. Lin et al. [26] evaluated the seismic performance of masonry arch walls strengthened with hybrid FRP materials, revealing substantial improvements in the bearing capacity, stiffness, and energy absorption. Notably, plate-type FRP reinforcements provided the highest energy dissipation under lateral loads and the greatest increase in load capacity under eccentric compression. Dan et al. [27] analyzed a five-story masonry structure and tower in seismic zones, addressing common damage, such as vertical cracking and steel member corrosion. Their reinforcement strategy involved vertical CFRP wrapping along the tower height and horizontal CFRP strips replacing corroded confining rings, enhancing overall stability. Gupta et al. [28] performed analytical and finite element simulations on unreinforced masonry domes retrofitted with CFRP or steel plates at the tension zones and supports, demonstrating significant reductions in the shell stresses ranging from 10% to 50%, depending on the retrofit configuration. Buyukkaragoz and Kopraman [29] compared the strength, ductility, and energy dissipation of masonry brick walls strengthened with lean and steel fiber-reinforced repair mortars of varying thicknesses. Finally, Hamdy et al. [30] conducted nonlinear numerical analyses of unreinforced masonry walls and vaults strengthened with various methods, including mortar, mild steel rods, FRP, ferrocement wire, and polyester mortar, concluding that FRP reinforcements yielded the greatest increases in the load-bearing capacity, with improvements of up to 190% for vaults.

The use of composite materials for reinforcing masonry domes has attracted increasing interest from researchers in recent years. However, despite this growing attention, the question of which regions of a dome benefit most from CFRP reinforcement has not been sufficiently investigated. This gap highlights the need for detailed studies. One of the most critical issues frequently observed in masonry domes after earthquakes, which weakens the load-bearing system, is damage to the body’s walls. In this study, a specialized “dome destruction apparatus,” capable of directly simulating this type of damage and presented for the first time in the literature, was designed and used in experimental investigations. The research analyzed the effect on the structural behavior of applying CFRP materials to different regions of the dome. In addition, the effects of the CFRP material in different regions were evaluated comparatively and it was determined in which regions the material provided the most efficient results. The study aims both to fill the gaps in the existing literature and to provide a scientific and technical resource to guide practitioners.

## 2. Materials and Methods

The mechanical properties of masonry domes were determined through standardized experimental procedures applied to masonry wall samples, individual masonry units, and traditional Khorasan mortar. These experiments were conducted at the Structural Mechanics Laboratory in the Department of Civil Engineering at Aksaray University, Turkey.

### 2.1. Characteristics of Khorasan Mortar

For centuries, Khorasan mortar and masonry units have served as fundamental construction materials in historic structures across Anatolia. In traditional masonry techniques, these units were bonded both horizontally and vertically with Khorasan mortar to form structural wall elements. The mortar was applied at both bed and head joints and typically consisted of approximately 40% lime, 40% stone powder, and 20% fine sand by volume.

To evaluate the compressive strength of Khorasan mortar, six cubic specimens measuring 50 mm × 50 mm × 50 mm were prepared and cured for 28 days. The compressive strength tests were conducted in accordance with EN 12390-3 [31].

The average compressive strength was calculated by dividing the peak load by the specimen’s cross-sectional area, yielding a mean value of 2.04 MPa. The flexural tensile strength of Khorasan mortar was determined in accordance with EN 12390-6 [32]. For this purpose, six prismatic mortar samples measuring 40 × 40 × 160 mm^3^ were prepared, cured for 28 days, and then subjected to mechanical testing. The flexural strength was calculated using the equation Rtf = PL/bd^2^, where Rtf denotes the flexural tensile strength (N/mm^2^), P is the maximum applied load (N), b is the width of the mortar (mm), d is the height (mm), and L is the length (mm). Based on this formulation, the average flexural tensile strength was found to be 0.79 MPa. The results of both the compressive and flexural tensile strength tests for the Khorasan mortar are presented in Table 1, where the compressive strength is indicated as KC and the flexural tensile strength as KT.

### 2.2. Characteristics of Stone

The mechanical properties of the andesite stone were determined through laboratory testing. The compressive strength was measured by subjecting six cubic specimens (50 mm × 50 mm × 50 mm) to uniaxial compression in accordance with EN 772-1:2011+A1:2015 [33]. The strength was calculated by dividing the maximum load sustained by each specimen by its loaded surface area. In parallel, the flexural tensile strength was evaluated using six prismatic specimens (40 mm × 40 mm × 160 mm), following the procedures outlined in EN 772-6 [34]. The average values obtained from the experimental program are presented in Table 2, where the compressive and flexural tensile strengths are denoted as AC and AT, respectively. The test results indicate an average compressive strength of 7.13 MPa and an average flexural tensile strength of 13.68 MPa.

### 2.3. CFRP Materials

CFRP was used in this study as a strengthening material to enhance the structural performance of masonry elements. Its high strength and low weight make it suitable for retrofitting historical structures without adding a significant load [7]. The main mechanical properties of the CFRP used are presented in Table 3.

CFRP was selected as the strengthening material due to its high mechanical strength, stiffness, and durability, which are essential for improving the seismic performance of historic masonry domes. Although CFRP is generally stiffer than other FRP types, its excellent strength-to-weight ratio allows the use of thin reinforcement layers, minimizing the additional weight and preserving the integrity of the original masonry. Moreover, the widespread use of CFRP in heritage conservation supports its suitability for this application [7,17].

The strengthening system employed unidirectional carbon fiber-reinforced polymer (CFRP) fabrics, with the fibers predominantly oriented in the horizontal direction. This specific fiber alignment was deliberately chosen to optimize the tensile resistance along the primary stress trajectories of the masonry dome. Such directional reinforcement enhanced the load-carrying capacity and seismic performance of the structure by efficiently transferring the tensile forces in the most critical zones. The material properties of the CFRP used in this study were determined based on the manufacturer’s technical documentation and validated through comparison with the data reported by Fırat and Eren [35].

### 2.4. Masonry Wall Specimens

**Figure 1:**
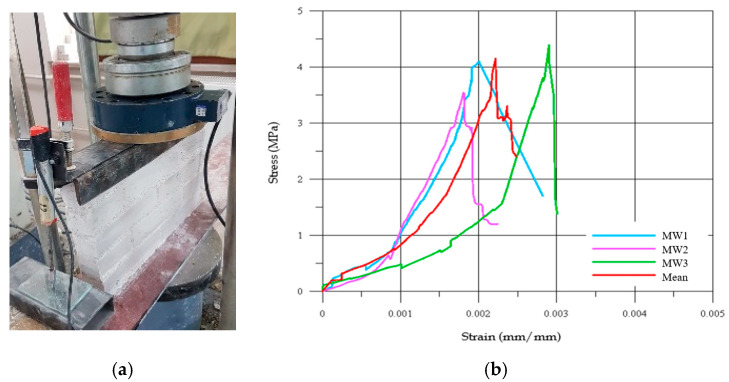
(**a**) Compressive strength test setup of masonry wall and (**b**) compressive stress–strain graphics.

To evaluate the compressive strength of the dome specimens in the experimental program, three masonry wall specimens with dimensions of 405 mm × 270 mm × 50 mm were subjected to uniaxial compression tests in accordance with TS EN 1052-1 [36]. The experimental setup used for the compressive loading of the masonry walls is illustrated in Figure 1a, and the corresponding stress–strain curves derived from the tests are presented in Figure 1b. The compressive strength test results, including the maximum compressive load (V), characteristic compressive strength (f), and Young’s modulus (E), are summarized in Table 4. The average nominal compressive strength of the wall specimens was determined to be 4.15 MPa. Furthermore, the ratio of the modulus of elasticity to the compressive strength (E/f) was calculated as 524, which is below the recommended limit of 1000 specified in TS EN 1996-1-1 [37]. This indicates a relatively low stiffness and a comparatively high deformation capacity of the masonry material.

## 3. Experimental Tests on Masonry Domes

### 3.1. Dome Specimens

In this study, the original dome dimensions were scaled down to ¼, considering the optimum geometric ratios of traditional domes commonly found in the eastern Mediterranean region. In line with the architectural typology frequently observed in historical examples, the dome’s height-to-diameter ratio was set to 1/2. In addition, an octagonal drum geometry was chosen, as it is the most commonly used shape in masonry domes.

In this study, CFRP was applied to different regions to strengthen the masonry domes. The strengthening interventions targeted the skirt region, the drum region, and the combined skirt–drum region of the dome. Four test specimens, each representing a different strengthening scenario, were tested in the laboratory, and corresponding numerical models were created for finite element analyses. The experimental results and numerical analysis outcomes were then evaluated comparatively.

**Figure 2:**
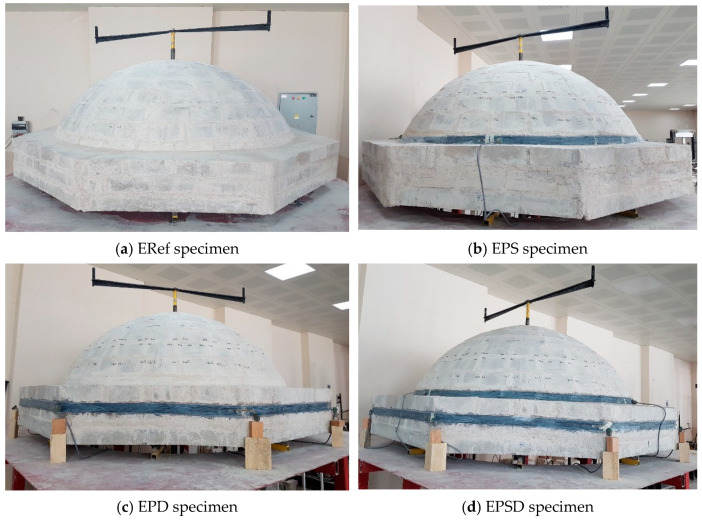
Dome specimens: (**a**) ERef (reference), (**b**) EPS (CFRP in the skirt area of the dome), (**c**) EPD (CFRP in the drum area of the dome), and (**d**) EPSD (CFRP in the skirt and drum areas of the dome).

Based on the analyses, the most appropriate intervention methods for repair and strengthening were determined by considering the crack widths and types of damage occurring in different regions of the dome. In this study, the reference sample, ERef, was not subjected to any reinforcement. In the EPS specimen, CFRP reinforcement was applied only to the skirt region, while in the EPD specimen, it was applied only to the drum region. The most comprehensive intervention was carried out on the ERSD specimen, where CFRP was applied to both the skirt and drum regions (Figure 2). This arrangement allowed for a detailed analysis of the effects of different regional reinforcement strategies on the structural performance.

The selection of the skirt and drum regions as strengthening zones was guided by previous post-earthquake damage assessments, which identified these areas as particularly vulnerable [15,23,38]. These regions are typically subjected to high tensile stresses during seismic events, resulting in circumferential cracking and potential instability. Strengthening these zones is therefore essential for improving the overall seismic resilience of masonry domes.

### 3.2. Test Setup and Instrumentation

In dome structures, separations in the body walls are a characteristic type of structural damage, resulting from horizontal thrust forces generated by the dome on its supporting walls. These forces develop due to the dome’s weight and seismic movements, causing the main walls to move outward. In domed structures, horizontal forces typically originate at the skirt level and increase toward the top of the wall. This can lead to openings between the load-bearing walls, jeopardizing the integrity of the structure [1].

Domes, due to their large size and heavy mass, require tall and robust supporting walls. However, their structural characteristics induce not only vertical forces from gravity but also significant horizontal thrusts, most pronounced at the edges and corners. The thrust begins at the drum area and progressively extends toward the skirt and apex. If the load-bearing walls are inadequately designed or structurally insufficient to resist these forces, they may experience separation, cracking, or even collapse over time. Such damage is generally caused by seismic activity or prolonged use of the structure.

**Figure 3:**
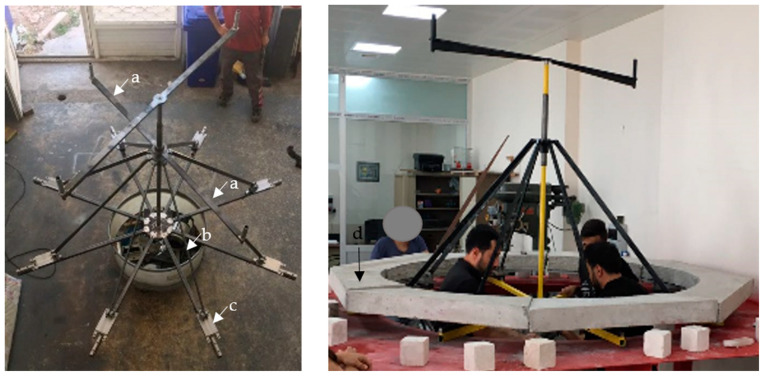
Dome destruction apparatus: (a) trapezoidal turning arm, (b) steel rods, (c) linear rail bearing, and (d) body walls.

In this study, a dome destruction apparatus was specifically designed and produced to simulate dome damage, for the first time in the literature (Figure 3). The device operates by rotating a trapezoidal arm at a constant speed in a clockwise direction. This movement drives eight steel rods downward at a 45-degree angle. The rods move along the axes of eight linear rail bearings to which they are attached, and together with the motion of the linear rail carriages, the walls of the structure slide outward along the direction of the dome pulley axes.

**Figure 4:**
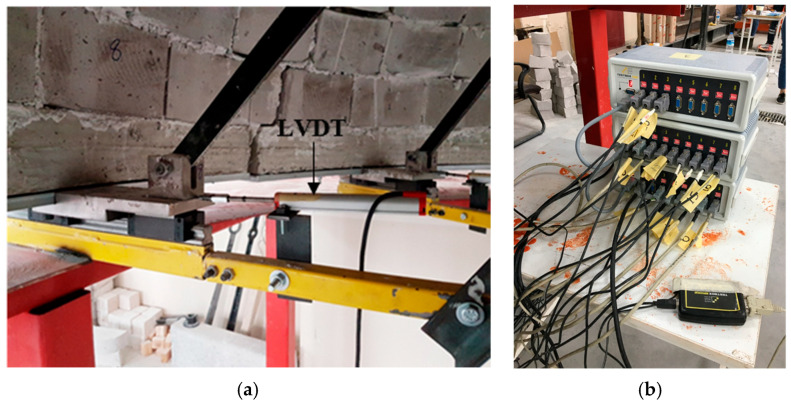
(**a**) Placement of LVDT and (**b**) data collection device.

S-type load cells, each with a capacity of 20 kN, were installed on the arms of the apparatus, arranged in pairs opposing each other to measure the forces. Linear variable differential transformer (LVDT) transducers were used to measure the displacements inside the dome. A total of eight LVDTs were installed on each linear rail bed to record the linear expansion and contraction of the dome walls. The placement of the LVDTs is shown in Figure 4. All the measurements from the load cells, LVDTs, and strain gauges were recorded at 250 data points per second by a data acquisition system and transferred to a computer (Figure 4).

The specially designed dome destruction apparatus replicates seismic-induced lateral thrust forces on the body walls with high fidelity by symmetrically applying outward forces at eight points around the dome base. Unlike traditional setups, which typically apply uniform vertical or horizontal loads, this device allows for a realistic simulation of the complex damage mechanisms characteristic of masonry domes under seismic action. Equipped with high-precision load cells and LVDT transducers, the apparatus provides accurate measurements of forces and displacements, yielding reliable experimental data. The number of applied forces can be adjusted according to the experimental requirements, enabling customized loading patterns to simulate different seismic scenarios and structural behaviors. This adaptability enhances the versatility and applicability of the apparatus, making it a valuable tool for evaluating strengthening interventions in historical masonry domes.

## 4. Numerical Analysis of Masonry Domes

In this study, the behavior of CFRP strengthening systems applied to different regions of masonry domes under seismic effects was investigated through finite element modeling. The numerical model was designed to simulate earthquake-induced damage in a manner consistent with the experimental setup. A three-dimensional finite element model of the domes was created using LUSAS V19.0 [39], employing 10-node tetrahedral solid elements with three degrees of freedom per node.

A macro-modeling approach was adopted to represent the masonry as a continuous, homogeneous medium with average mechanical properties. This technique, as suggested by Lourenço [40,41], allows for the efficient analysis of large-scale masonry structures without modeling individual bricks and mortar joints, capturing the global behavior while maintaining computational feasibility.

In the finite element model, the masonry dome was represented using 3D solid elements, while the CFRP reinforcement was modeled as 2D surface elements attached to the external faces of the structure. A perfect bond condition was assumed at the interface between the CFRP and masonry, implying no slip or separation. This simplification has been commonly used in similar studies and was deemed acceptable due to the good agreement between the numerical and experimental results. Nevertheless, more detailed interface modeling (e.g., using cohesive elements) could be considered in future studies to capture potential debonding or slip effects.

The CFRP and epoxy components were modeled as a single isotropic, homogeneous material. Although CFRP materials are generally anisotropic, the reinforcement in this study was applied only to areas subjected to unidirectional tensile stresses. Therefore, the materials’ properties in the transverse directions (such as Poisson’s ratio or elasticity modulus) were considered negligible. This modeling choice simplified the analysis without significantly affecting the results’ accuracy. Furthermore, the literature, such as Hollaway and Teng [42], indicates that the interaction between the FRP and epoxy may lead to a limited reduction in strength; however, this effect is generally negligible in terms of the overall structural behavior. Therefore, modeling the CFRP and epoxy as a single homogeneous composite material is a widely accepted and appropriate approach that balances computational efficiency with model accuracy.

The nonlinear material behavior was modeled using the Drucker–Prager failure criterion, which is suitable for quasi-brittle materials such as masonry. The analysis proceeded incrementally, with loading continuing until either convergence failure or material failure occurred. No predefined displacement or force termination conditions were applied; instead, the model was allowed to reach natural termination based on numerical instability or loss of load-carrying capacity. This approach ensured that the full progression of damage and ultimate failure modes were realistically captured.

**Figure 5:**
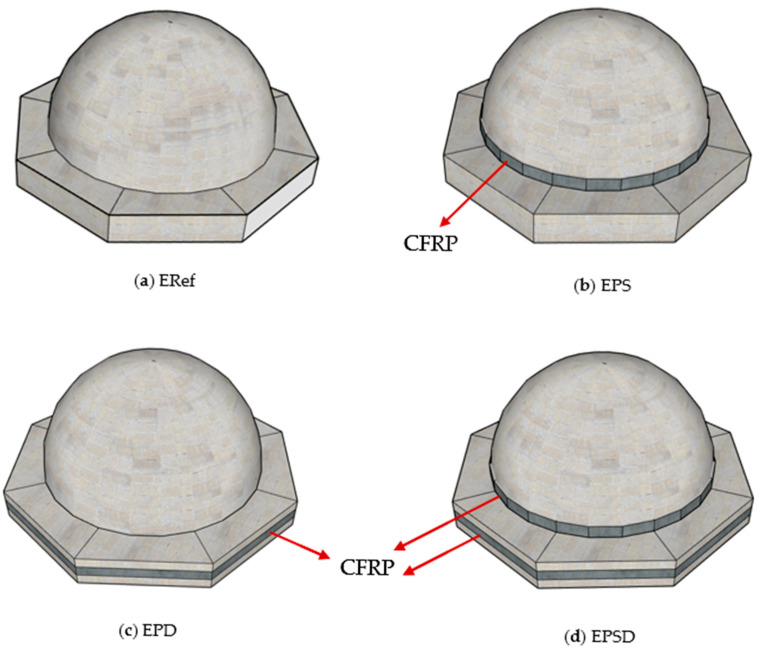
Materials used in modeling of masonry dome.

**Figure 6:**
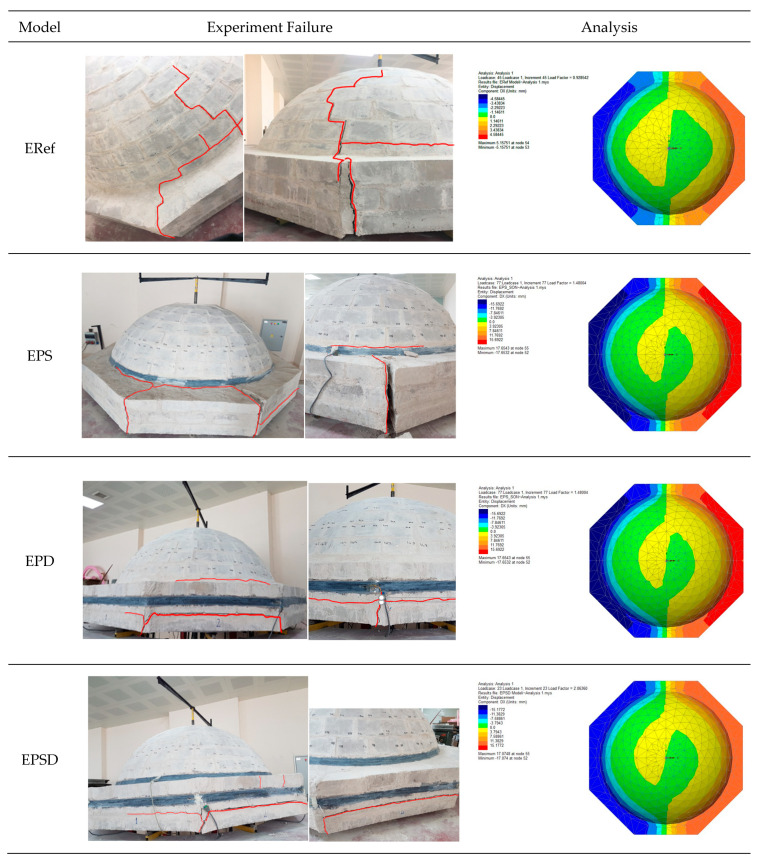
Comparison of finite element simulation and experimental failure modes of masonry dome specimens.

Understanding the structural behavior of historical structures and accurately modeling the performance of load-bearing systems are crucial for the success of conservation and reinforcement efforts. In this study, the reinforcement materials applied to different parts of the dome were analyzed using a finite element method and evaluated comparatively in terms of load and displacement. According to the analysis, the ERef model exhibited a very rigid behavior, with a maximum displacement of 5.15 mm under a load of 0.92 kN. The EPS model, in contrast, showed a displacement of 17.65 mm under a maximum load of 1.48 kN, indicating a lower structural integrity compared to the other reinforced models. The EPSD model was displaced 17.07 mm under a maximum load of 2.06 kN. The EPD model demonstrated that this reinforcement effectively increased the structural rigidity, with a displacement of 16.29 mm under a maximum load of 2.86 kN. Overall, examining the displacement data and the corresponding graphs from all the models revealed that the EPD model exhibited the highest endurance throughout the dome structure. Table 5 shows the material properties defined for the numerical models. Figure 5 illustrates the materials used in the modeling of the masonry dome, while Figure 6 compares the finite element simulation and experimental failure modes of the dome specimens.

## 5. Results and Discussion

Strengthening the masonry domes with the CFRP material (Table 6), an innovative reinforcement method, resulted in significant improvements in both the bearing capacity and maximum displacement.

In this study, the performance of CFRP reinforcement in different regions of dome structures was evaluated. The reference specimen, ERef, achieved a bearing capacity of 0.92 kN and a maximum displacement of 5.08 mm, indicating a low load-bearing capacity and limited deformation. In the EPS specimen, the bearing capacity increased to 1.48 kN—a 160.87% increase—but the high horizontal displacement of 17.49 mm indicates that this specimen has limited overall structural performance. This suggests that the EPS reinforcement is effective in less durable, low-bearing-capacity areas of the dome, but insufficient to enhance the overall strength of the structure.

The EPD specimen exhibited a bearing capacity of 2.86 kN, an increase of 310.87%, with a maximum horizontal displacement of 16.03 mm. This demonstrates that reinforcing the drum region provides a high-performing solution, significantly increasing the bearing capacity while limiting deformation in the weaker, deformation-prone areas. The EPSD specimen achieved a bearing capacity of 2.06 kN, an increase of 223.91%, with a maximum displacement of 17.05 mm. This indicates that strengthening both the skirt and drum regions provides a balanced improvement, controlling deformation while increasing the load capacity.

**Figure 7:**
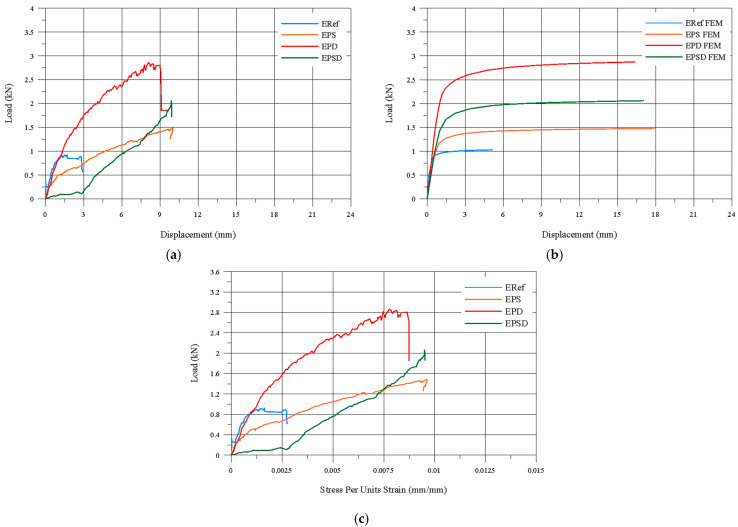
(**a**) Experimental load–vertical displacement graph, (**b**) FEM numerical load–vertical displacement graph, and (**c**) experimental load–vertical strain graph.

**Figure 8:**
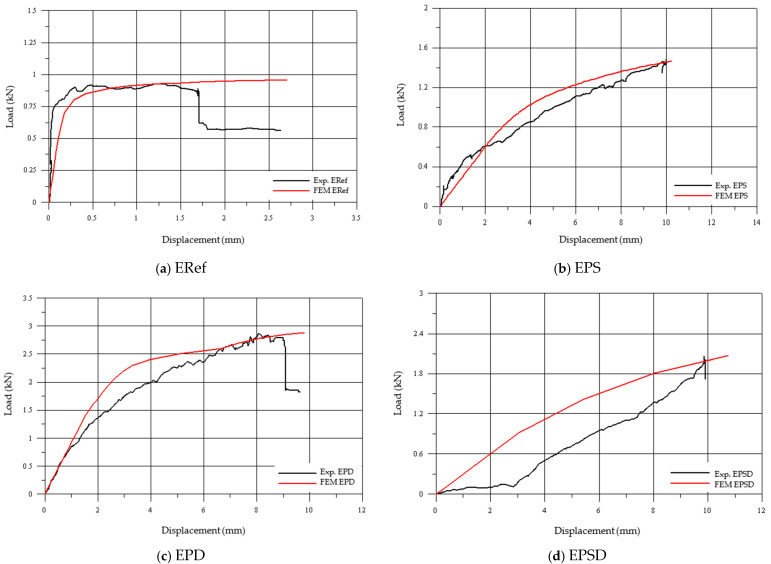
Comparison of experimental and numerical analysis results: (**a**) ERef, (**b**) EPS, (**c**) EPD, and (**d**) EPSD.

Overall, CFRP reinforcement in the different regions significantly enhanced the bearing capacity and controlled deformation, showing that each strengthening method can be optimized according to the specific structural requirements. The stress-per-unit-strain values further confirmed that the reinforced specimens had a greater deformation capacity compared to the unreinforced ERef specimen. The EPS specimen showed the highest value (0.00956 mm/mm) with reinforcement applied to the skirt, while the EPD specimen achieved 0.00927 mm/mm with reinforcement at the drum. The EPSD specimen reached 0.00949 mm/mm, indicating that strengthening both zones did not provide additional benefit. Figure 7 presents three different graphs used to compare the experimental and numerical results: (a) experimental load–vertical displacement, (b) numerical load–vertical displacement, and (c) experimental load–vertical strain. Figure 8 shows a direct comparison of the experimental and numerical results.

Although the numerical model closely matched the experimental results, minor discrepancies in the displacement values were observed. These variations can be attributed to several factors, including assumptions made in the finite element model, such as idealized boundary conditions, homogenized material properties, and the inability to fully capture the heterogeneous nature of masonry. Additionally, experimental uncertainties, including measurement errors, material inconsistencies, and imperfections in specimen fabrication, may have contributed to the differences. Despite these limitations, the strong correlation between the numerical and experimental results confirms the validity and reliability of the model for representing the structural behavior of masonry domes.

For the preservation of cultural heritage, it is essential that strengthening interventions follow the principles of minimal intervention and reversibility to protect original structures. The CFRP reinforcement applied in this study was localized to the critical regions, such as the dome skirt and drum, minimizing the impact on the original fabric. These interventions significantly improved the seismic performance of the domes by enhancing its structural resilience and integrity under earthquake loading. Additionally, the selected CFRP material can be removed or replaced in the future without damaging the historic masonry; this ensures that the interventions remain reversible and that the integrity of the historic structure is preserved.

Unidirectional CFRP composite material is inherently anisotropic; however, in this study, isotropic material properties were used. This simplification may have affected the local stress distribution within the FRP material, but it adequately represented the macro-scale structural response of the strengthened masonry domes. Detailed local stress predictions within the FRP material should consider anisotropic modeling if such information is required.

## 6. Conclusions

This study investigated the effects of CFRP strengthening on the bearing capacity and deformation behavior of dome structures in the skirt, drum, and skirt–drum regions through experimental and numerical analyses. The analyses, conducted on a reference specimen and three specimens retrofitted with CFRP in the different dome regions, allowed for a comparative evaluation of the impact of strengthening on the structural performance. The results show that CFRP reinforcement is an effective method for increasing the bearing capacity and improving the deformation behavior in various parts of domes. The main findings of this study are summarized below:

- The analysis showed that the CFRP drum (EPD) specimen carried 1.4 times more of a load than both the CFRP skirt and drum (EPSD) specimen and the CFRP skirt (EPS) specimen. This indicates that CFRP applications are more effective in the drum region and can play a key role in increasing the bearing capacity. In addition, the CFRP reinforcement was found to be more efficient in the angular regions of the dome (drum and skirt) compared to the circular regions (skirt–drum), enhancing the structure’s load-carrying capacity. It was also determined that reinforcing the angular regions is easier and more practical than applying CFRP to the circular regions.
- Evaluation of damage formation in the domes showed that cracks in the CFRP-reinforced specimens occurred mainly in the tensile zones. The cracks were particularly concentrated in the skirt region, the upper part of the body wall, and the corners of the domes, with the crack openings varying depending on the reinforcement zone. In this context, the EPSD specimen experienced the least damage, while the EPS specimen showed the highest number of cracks and overall damage. In the EPSD specimen, reinforcing both the skirt and drum regions limited the dome’s tendency to open and provided more effective protection of structural integrity.
- Evaluation of damage in the drum area showed that the cracks in the skirt corners of the EPD specimen were larger and more pronounced compared to the ERD specimen. Conversely, although the crack openings in the upper part of the body wall were larger in the ERD specimen than in the EPD specimen, crack propagation toward the upper parts of the domes was more effectively limited in the ERD specimen. This indicates that not only the crack density and spacing, but also the direction of crack propagation, vary significantly depending on the location of the retrofit application.
- Comparison of the experimental results with the numerical analysis obtained using the finite element method showed that the displacement values increased in parallel with the load in both models. The displacement values, particularly at maximum load levels, were found to be very close. Furthermore, the load–displacement curves from the experimental and numerical analyses largely overlapped, demonstrating that the developed finite element model successfully captured the experimental behavior.
- This study focused on experimental and numerical analyses, but future research should also consider analytical solution methods to provide a more comprehensive evaluation of the effects of different modeling approaches. Additionally, by varying the geometric dimensions of the CFRP materials, the impact of dimensional differences on structural performance can be investigated, allowing for determination of the optimal strengthening parameters for dome behavior.
- While the EPSD strengthening method provided balanced structural improvement, practical factors, such as accessibility, as well as increased labor and material costs, must also be considered in real-world applications.
- While the study confirms the effectiveness of CFRP strengthening of the drum region, limitations, such as scalability to full-scale domes, long-term durability under environmental conditions, and cost implications, should be considered. Further research involving full-scale experiments and long-term monitoring is needed to fully validate the practical application of this method to heritage structures.
- This study highlights the effectiveness of CFRP materials for strengthening historic dome structures, showing that the bearing capacity can be increased by reinforcing the drum region. The results indicate that CFRP applications are particularly efficient in angular regions, providing a reliable solution for strengthening domes. Furthermore, this study offers a solid foundation for future research and serves as a guiding resource for researchers in the field.
- Future research will focus on dynamic testing to evaluate the seismic performance of masonry domes strengthened with CFRP materials. Additionally, hybrid CFRP techniques and regional reinforcement optimization will be explored to identify the most effective strengthening methods. Their long-term durability under environmental conditions will also be assessed to develop sustainable intervention strategies. These efforts aim to contribute significantly to the preservation of historic domes.

## Figures and Tables

**Table 1:** Compressive and flexural tensile strength results of Khorasan mortar.

Series	Maximum Loading (kN)	Compressive Strength (N/mm^2^)	Series	Maximum Loading (kN)	Flexural Tensile Strength (N/mm^2^)
KC1	5.45	2.18	KT1	0.35	0.88
KC2	5.07	2.03	KT2	0.30	0.75
KC3	4.79	1.92	KT3	0.28	0.70
KC4	5.25	2.10	KT4	0.34	0.85
KC5	4.87	1.95	KT5	0.30	0.75
KC6	5.17	2.07	KT6	0.33	0.83
Mean	5.10	2.04		0.32	0.79
±SD	0.25	0.10		0.027	0.07

KC1–KC6: Compressive strength of mortar. SD denotes standard deviation. KT1–KT6: Flexural tensile strength of mortar.

**Table 2:** Compressive and flexural tensile strength results of stone.

Series	Maximum Loading (kN)	Compressive Strength (N/mm^2^)	Series	Maximum Loading (kN)	Flexural Tensile Strength (N/mm^2^)
AC1	18.34	7.34	AT1	6.15	15.38
AC2	18.21	7.28	AT2	5.87	14.68
AC3	18.17	7.27	AT3	5.65	14.13
AC4	17.79	7.12	AT4	5.18	12.95
AC5	17.52	7.01	AT5	5.12	12.80
AC6	16.91	6.76	AT6	4.85	12.13
Mean	17.82	7.13		5.47	13.68
±SD	0.54	0.22		0.50	1.25

AC1–AC6: Compressive strength of stone. SD denotes standard deviation. AT1–AT6: Flexural tensile strength of stone.

**Table 3:** Properties of the reinforcement materials [35].

System	Thickness (t), mm	Yield Strength (MPa)	Strain at Ultimate (µ-Strain)	Modulus of Elasticity, GPa
CFRP (Unidirectional)	0.131	4300	0.018	234
Epoxy	-	12,500	0.009	3.8

**Table 4:** Compressive strength of masonry walls.

Specimens	Max. Load, V (kN)	Compressive Strength, f (MPa)	Young’s Modulus, E (MPa)	Ratio, E/f
MW1	81.80	4.09	2038	582
MW2	70.60	3.53	1947	556
MW3	87.80	4.39	1514	433
Mean	80.07	4.00	1833	524
±SD	8.73	0.44	279.97	79.57

**Table 5:** Defined material properties of numerical models.

Materials	Properties	Values
Masonry Wall	Elasticity module (MPa)	1850
	Poisson’s ratio	0.2
	Cohesion (MPa)	2.5
	Internal friction angle	25
	Plastic deformation	0.001
CFRP + Epoxy	Elasticity module (MPa)	234,000
	Poisson’s ratio	0.3
	Uniaxial yield stress (MPa)	4300
	Plastic deformation	0.018

**Table 6:** Experimental test results.

Experiment Name	Max. Load (kN)	Increase ^a^ (%)	Displacement (mm)	Stress per Unit Strain (mm/mm)
ERef Experiment	0.92	0	5.08	0.00265
EPS Experiment	1.48	160.87	17.49	0.00956
EPD Experiment	2.86	310.87	16.03	0.00927
EPSD Experiment	2.06	223.91	17.05	0.00949

^a^ Percentage increase in the maximum load according to the ERef specimen of the models.

## Data Availability

The original contributions presented in this study are included in the article. Further inquiries can be directed to the corresponding author.
